# Superiority of the modified Tönnis angle over the Tönnis angle in the radiographic diagnosis of acetabular dysplasia

**DOI:** 10.3892/etm.2014.2009

**Published:** 2014-10-08

**Authors:** LIANGGUO FA, QING WANG, XIANGXING MA

**Affiliations:** 1 Department of Medical Radiology, The Second Hospital of Shandong University, Jinan, Shandong 250033, P.R. China; 2 Department of Medical Radiology, Qilu Hospital of Shandong University, Jinan, Shandong 250012, P.R. China

**Keywords:** center-edge angle, center-medial-edge angle, Tönnis angle, modified Tönnis angle, acetabular sourcil, pelvic radiograph

## Abstract

The aim of this study was to evaluate the limitations of the Tönnis angle as one of the most commonly used parameters in the diagnosis of acetabular dysplasia, and to explore the feasibility of the modified Tönnis angle in the diagnosis of acetabular dysplasia. A total of 224 patients (120 females and 104 males) with 448 hips, aged between 15 and 83 years (median, 45.0 years), were selected for the measurement of the center-edge (CE) and Tönnis angles. To evaluate the relative position of the medial edge of the acetabular sourcil, a new parameter, known as the center-medial-edge (CME) angle, was designed. As an improvement of the Tönnis angle, a new angle preliminarily termed the modified Tönnis angle was created. In addition, the degree of clarity of the medial edge of the acetabular sourcil on radiograph was evaluated, and the hips were divided into the clear-edge and blurred-edge groups. The hips belonging to the blurred-edge group could not be used for Tönnis angle measurements. All measurements were performed digitally using the tool of the picture-archiving communication system. Among the 448 acetabular sourcils, 142 had a blurred medial edge (31.7%). The mean value of the CME angle was 37.94°, with a range of 21.76–63.99°. The 95% prediction interval of the modified Tönnis angle was estimated to be −6.39 to 11.73°. The correlation coefficients were −0.838 between the CE and Tönnis angles, 0.889 between the Tönnis and modified Tönnis angles and −0.905 between the CE and modified Tönnis angles. In conclusion, the modified Tönnis angle can substitute for the Tönnis angle without joint space narrowing and subluxation of the hip, particularly when the Tönnis angle cannot be measured due to a blurred medial edge of the acetabular sourcil on pelvic radiograph.

## Introduction

Acetabular dysplasia is a relatively common abnormality of the anatomy of the acetabulum, whose prevalence in adults varies across studies from 1–15% (1–4). The disease is usually undiagnosed until the appearance of symptoms during the second or third decade of a patient’s life. Dysfunction, pain and limpness are the initial symptoms of acetabular dysplasia in adults (5). In total, ~20–50% of adults develop arthritis as a result of subluxation or dysplasia of the hip (6). Adults with acetabular dysplasia usually have a shallow or deformed acetabulum, occasionally with luxation or subluxation of the hip (7). Patients with persistent acetabular dysplasia and subluxation are at high risk of osteoarthritis (OA) (8,9). The lack of early diagnosis and treatment for acetabular dysplasia can lead to OA in adults (10). At present, the imaging diagnosis of acetabular dysplasia typically depends on radiographic evaluation. Certain parameters, such as the center-edge (CE) and Tönnis angles, are the most commonly used measurements of acetabular dysplasia (7,11,12).

The Tönnis angle measures the weight-bearing surface of the acetabulum, otherwise known as the acetabular sourcil. More precisely, the acetabular sourcil represents an area of subchondral osseous condensation in the acetabular roof (7). The Tönnis angle is formed between a horizontal line and a tangential line extending from the medial edge to the lateral edge of the acetabular sourcil; however, in clinical practice, the medial edges of certain acetabular sourcils on hip radiographs are not distinct (13), resulting in the inaccuracy or impossibility of Tönnis angle measurement. In addition, the relative positions of the medial edges of the acetabular sourcils may have a number of individual differences, which may affect the accuracy of the Tönnis angle measurement. The aim of the present study was to evaluate the clarity and relative position of the medial edge of the acetabular sourcil on hip radiographs and to attempt to improve the Tönnis angle.

## Materials and methods

### Patients

Conventional anterior-posterior pelvic radiographs of patients, obtained between December 2012 and February 2013, were extracted from the picture-archiving communication system in The Second Hospital of Shandong University (Jinan, China). The exclusion criteria comprised narrowing of the hip joint space, subluxation and luxation of the hip joint, osteonecrosis of the femoral head, acetabular and femoral fracture, hip tumor and total hip arthroplasty. According to the criteria of Siebenrock *et al* (14), further assessments were made of the anterior-posterior pelvic radiographs showing the alignment of the tip of the coccyges with the middle of the symphysis pubis and the distance between the sacrococcygeal joint and the symphysis pubis (<32 mm in males and <47 mm in females). With these criteria, 224 patients (120 females and 104 males) with 448 hips, aged between 15 and 83 years (median, 45.0 years), were selected for this study. All procedures were approved by the Ethics Committee of The Second Hospital of Shandong University. Informed consent was obtained from all patients or their families.

### Measurements on radiographs

**Figure 1:**
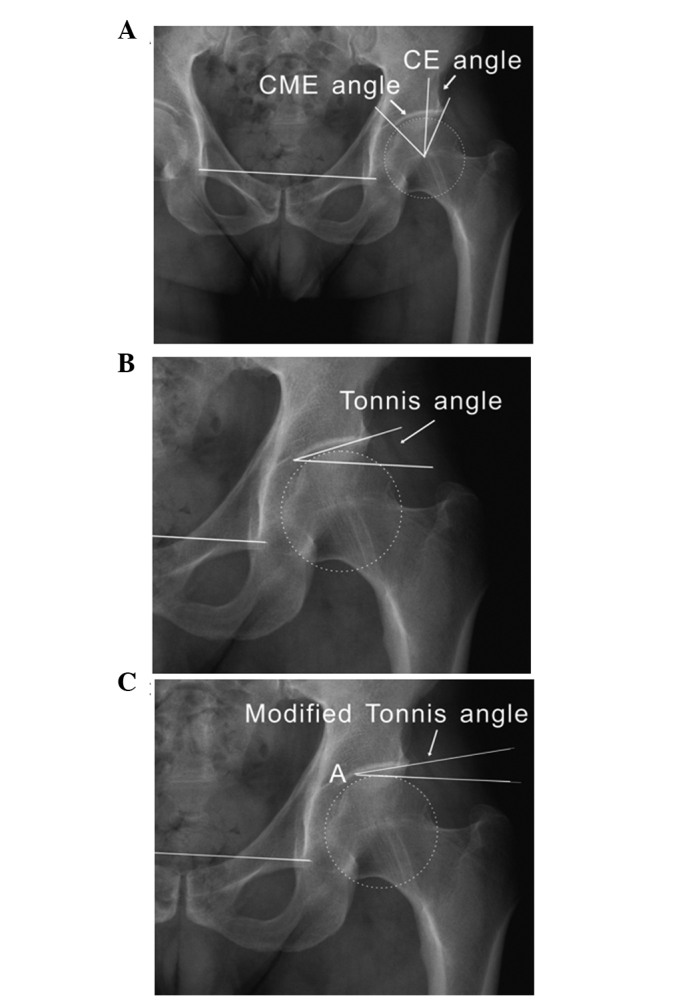
Measurements of angles. (A) Center-edge (CE) angle and center-medial-edge (CME) angle. The horizontal plane of the pelvis was determined by a horizontal line connecting the inferior boundaries of the two teardrops (teardrop to teardrop). The CE angle is the angle between a vertical line perpendicular to the horizontal plane of the pelvis and an oblique line connecting the femoral head center to the lateral acetabulum margin. The CME angle is the angle between the vertical line as described above and an oblique line connecting the femoral head center to the medial margin of the acetabular sourcil. (B) The Tönnis angle, formed by a line parallel to the horizontal plane of the pelvis and a tangential line extending from the medial edge to the lateral edge of the acetabular sourcil. (C) The modified Tönnis angle. A parallel line to the horizontal plane of the pelvis touching the vertex of the femoral head is drawn. This line intersects with the acetabular sourcil at a point marked ‘A’. The angle between the parallel line and a line connecting point A to the lateral margin of the acetabulum shows the modified Tönnis angle.

To measure the CE angle, the horizontal plane of the pelvis was firstly determined by drawing a horizontal line along the inferior boundaries of the two teardrops. A vertical line perpendicular to the horizontal plane of the pelvis was then drawn as described above (teardrop to teardrop), crossing the center of the femoral head. Finally, an oblique line from the center of the femoral head was drawn out to the lateral margin of the acetabulum. The angle between the vertical line and the oblique line showed Wiberg’s CE angle (Fig. 1A).

The Tönnis angle was formed by a line parallel to the horizontal plane of the pelvis (teardrop to teardrop) touching the medial edge of the weight-bearing portion of the acetabulum (the ‘sourcil’) and a tangential line extending from the medial edge to the lateral edge of the acetabular sourcil (Fig. 1B).

To evaluate the relative position of the medial edge of the acetabular sourcil, a new parameter known as the center-medial-edge (CME) angle was designed. The CME angle was the angle between the vertical line through the femoral head center, as described above, and the line that was tangential to the femoral head center and the medial margin of the acetabular sourcil (Fig. 1A).

As an improvement of the Tönnis angle, a new angle, preliminarily termed the modified Tönnis angle, was created. The measure this angle, a line parallel to the horizontal plane of the pelvis was drawn as described above (teardrop to teardrop), touching the vertex of the femoral head. This line intersected with the acetabular sourcil at a point marked ‘A’. A line originating from point A was then drawn out to the lateral extent of the weight-bearing portion of the acetabulum. The angle between these two lines indicated the modified Tönnis angle (Fig. 1C). If the lateral edge of the acetabular sourcil was below the parallel line, the value of the modified Tönnis angle was negative.

**Figure 2:**
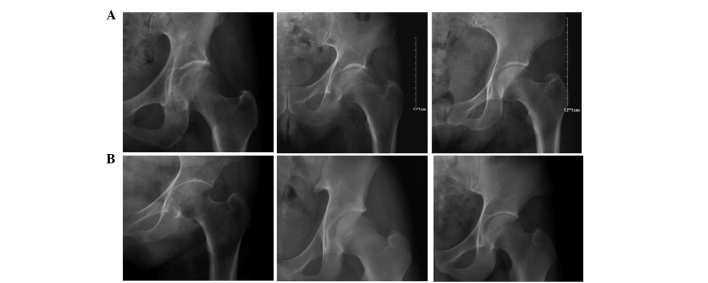
Radiographs showing (A) acetabular sourcils with clear medial edges, which can be used to measure the Tönnis angle, and (B) hips assigned to the blurred-edge group, in which the medial edges of the acetabular sourcils are indistinct.

According to the clarity degree of the medial edge of the acetabular sourcil on radiograph, the hips were divided into the clear-edge (Fig. 2A) and blurred-edge (Fig. 2B) groups. The hips belonging to the blurred-edge group could not be used for Tönnis angle measurements. All measurements were performed digitally using the Huahai MedPACS picture-archiving communication system (Huahai Medical Info-Tech Co., Ltd., Xi’an, China).

### Statistical analysis

All statistical analyses were performed using SPSS 18.0 for Windows (SPSS, Inc., Chicago, IL, USA). Statistical analysis of the type of acetabular sourcil based on the medial edge clarity was made. The Gaussianity of various parameters was graphically revealed using frequency polygons. Associations between any two of the CE angle, the Tönnis angle and the modified Tönnis angle were evaluated using Pearson’s coefficient of correlation. The association between the CME and CE angles was also evaluated. Differences were considered significant when P<0.01.

## Results

### Hips belonging to the blurred-edge group cannot be used for Tönnis or CME angle measurements

To measure the values of the CE, CME, Tönnis and modified Tönnis angles, 448 hips were analyzed. Among these hips, 142 (31.7%) were assigned to the blurred-edge group and 306 (68.3%) had clear medial sourcil edges that were used to measure the Tönnis and CME angles. All 448 hips were used for CE and modified Tönnis angle measurement. The mean CME angle was 37.94° with a range of 21.76–63.99° [n=306; 95% confidence intervals (CI), 37.22–38.66°; standard deviation, 6.41]. The mean modified Tönnis angle was 2.67° (n=448; 95% CI, 2.24–3.10°; standard deviation, 4.62), with the 95% prediction interval being estimated to be −6.39 to 11.73°. These data demonstrated that hips in the blurred-edge group could not be used for Tönnis or CME angle measurements, but the limitation did not exist in the measurements of the CE and modified Tönnis angles.

### CME angle values vary across a large range

**Figure 3:**
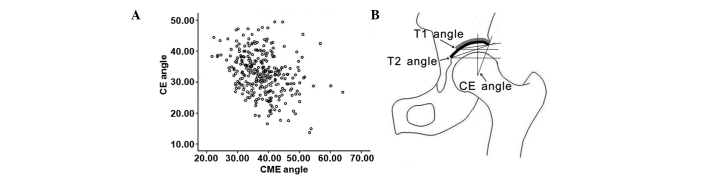
(A) Scatter plot showing weak association between the center-edge (CE) and center-medial-edge (CME) angles. (B) Diagram illustrating a possible situation in which two hips with the same CE angle value have two different acetabular sourcils with different relative medial edge positions, resulting in two different Tönnis angles (T1 and T2).

To evaluate the correlation between the CE and CME angles, a scatter plot was drawn. According to the scatter plot, the points representing the CE and CME angles were distributed in the coordinate without any order, suggesting weak association between the two angles (Fig. 3A). It is possible that a situation could exist in which two hips with the same CE angle value could have two acetabular sourcils with marked differences between the relative medial edge positions; this would generate two notably different Tönnis angles, thus affecting the correlation between the CE and Tönnis angles (Fig. 3B). These data indicated that the relative positions of the medial edges of the acetabular sourcils in hips could vary across a large range, which could decrease the correlation coefficient between the CE and Tönnis angles to a certain degree.

### Theoretically, the value of angle B is only affected by the radii of the femoral head and acetabulum

**Figure 4:**
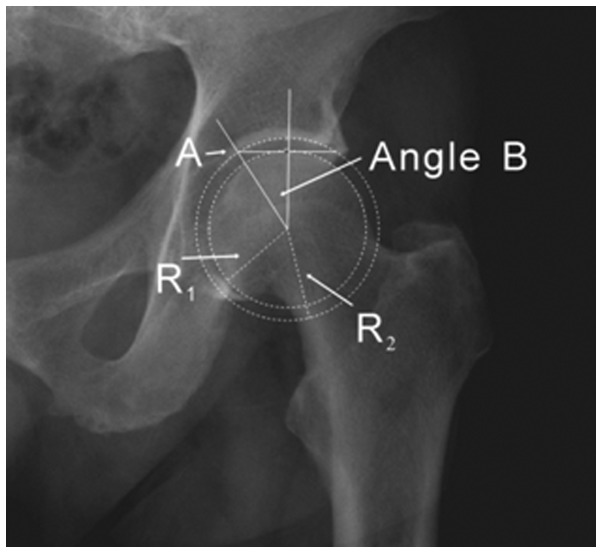
Radiograph showing the relative position of point A. Point A is used to measure the modified Tönnis angle, and the relative position of point A is determined by angle B. R_1_ and R_2_ represent the radii of the femoral head and acetabulum, respectively. Theoretically, cosB = R1/R2; therefore, the relative position of point A can only be affected by the radii of the femoral head and acetabulum.

To improve the Tönnis angle, the modified Tönnis angle was created, which was formed by two lines that intersected at a point marked ‘A’ (Fig. 1C). Point A was used to measure the modified Tönnis angle, and the relative position of point A was determined by angle B (Fig. 4). R_1_ and R_2_ represented the radii of the femoral head and acetabulum, respectively. Theoretically, cosB is equal to R_1_/R_2_; therefore, the relative position of point A can only be affected by the radii of the femoral head and acetabulum. Compared with the medial edge of the acetabular sourcil, point A was clear in all patients, and its relative position was more stable. These data suggested that point A could rarely negatively affect the measurement of the modified Tönnis angle.

### Positive or negative correlations exist between any two of the CE, Tönnis and modified Tönnis angles

**Figure 5:**
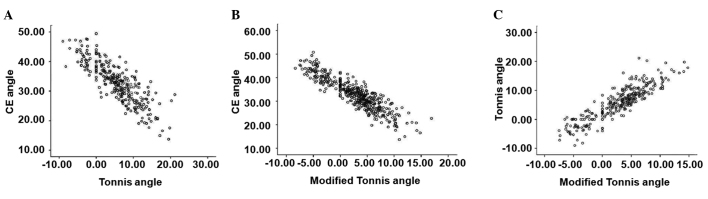
Scatter plots showing correlations between (A) the center-edge (CE) and Tönnis angles; (B) the CE and modified Tönnis angles and (C) the Tönnis and modified Tönnis angles.

To evaluate whether correlations existed between any two of the CE, Tönnis and modified Tönnis angles, three scatter plots were drawn. The correlation coefficients were −0.838 between the CE and Tönnis angles (n=306, P<0.01), 0.889 between the Tönnis and modified Tönnis angles (n=306, P<0.01), and −0.905 between the CE and modified Tönnis angles (n=448, P<0.01) (Table I and Fig. 5). These data demonstrated that strong negative or positive correlations existed between any two of the CE, Tönnis and modified Tönnis angles, and the correlation between the CE and modified Tönnis angles was the strongest.

## Discussion

Wiberg’s CE angle and the Tönnis angle are commonly used to measure hip dysplasia. A CE angle value of <20° indicates hip dysplasia. Values of 20–25° are considered borderline dysplasia, representing hips that are at the lower normal limits in terms of femoral head coverage, but not quite considered to have uncovering (15). The Tönnis angle is also known as the acetabular roof angle of Tönnis, the weight-bearing acetabular index, Lequesne’s acetabular index, the acetabular roof obliquity and the horizontal toit externe angle (7,16,17). This angle measures the weight-bearing surface of the acetabulum or sourcil. A Tönnis angle >13° is considered to indicate hip dysplasia (18). The intra- and interobserver reproducibility of the Tönnis and CE angles are reported to be satisfactory (19–21), indicating that they are reliable measurements in clinical practice.

The obliquity of the acetabular sourcil is directly indicated by the Tönnis angle, and the clarity and relative position of the medial edge of the acetabular sourcil on radiographs can affect the measurement of the Tönnis angle. In this study, 142 sourcils had blurred medial edges, accounting for ~31.7% of all sourcils; these could not be used for Tönnis angle measurements. One reason is that boney confluens may exist between a thickened medial wall caused by heterotopic bone in the cotyloid fossa and the medial margin of the acetabular sourcil (13). Another reason is that osteopenia in acetabular subchondral bone was present on plain radiographs, due to bone marrow edema (22). Evidently, the measurement of the Tönnis angle occasionally became impossible on account of the blurred medial edge of the sourcil, and could be influenced by the relative position of the sourcil’s medial edge; these could be considered to be limitations of the Tönnis angle in the diagnosis of acetabular dysplasia.

In this study, all 448 hips, including 142 hips that had blurred medial edges of the acetabular sourcils, were successfully evaluated by the modified Tönnis angles. The upper limit of the 95% reference range for the modified Tönnis angle was 11.73°, and the diagnosis of acetabular dysplasia could be made when a modified Tönnis angle was >12°. Additionally, strong negative or positive correlations existed between any two of the CE, Tönnis and modified Tönnis angles, and the correlation between the CE and modified Tönnis angles was the strongest. It is, however, noteworthy that there are certain adverse factors, including joint space narrowing (JSN) and subluxation of the hip, which can lead to inaccurate measurement of the modified Tönnis angle. The existence of JSN can cause a decrease in the value of the modified Tönnis angle. In addition, subluxation of the hip can increase the modified Tönnis angle or make the measurement of the modified Tönnis angle impossible.

In conclusion, this study demonstrated that the modified Tönnis angle was a feasible and available parameter for radiographic evaluation of acetabular dysplasia, and could substitute for the Tönnis angle without JSN or subluxation of the hip, particularly when the Tönnis angle could not be measured due to a blurred medial edge of the acetabular sourcil on pelvic radiograph. Further studies are necessary to determine the reliability of the modified Tönnis angle as a diagnostic parameter of acetabular dysplasia.

## Figures and Tables

**Table I:** Correlation coefficients between any two of the CE, Tönnis, modified Tönnis and CME angles.

Angle	Statistical analysis	CE angle	Tönnis angle	Modified Tönnis angle	CME angle
CE angle	Pearson correlation	1	−0.838^a^	−0.905^a^	−0.309^a^
	P-value (two-tailed)		<0.001	<0.001	<0.001
	N	448	306	448	306
Tönnis angle	Pearson correlation	−0.838^a^	1	0.889^a^	0.635^a^
	P-value (two-tailed)	<0.001		<0.001	<0.001
	N	306	306	306	306
Modified Tönnis angle	Pearson correlation	−0.905^a^	0.889^a^	1	0.316^a^
	P-value (two-tailed)	<0.001	<0.001		<0.001
	N	448	306	448	306
CME angle	Pearson correlation	−0.309^a^	0.635^a^	0.316^a^	1
	P-value (two-tailed)	<0.001	<0.001	<0.001	
	N	306	306	306	306

a Correlation is significant at the 0.01 level (two-tailed).

CE, center-edge; CME, center-medial-edge.
